# Supplementary material for: Bayesian refinement of protein structures and ensembles against SAXS data using molecular dynamics
Source: PLoS Comput Biol. 2017 Oct 18;13(10):e1005800. doi: 10.1371/journal.pcbi.1005800 (PMC5662244; doi:10.1371/journal.pcbi.1005800)
Supplement: S1 Archive — (BZ2) [file pcbi.1005800.s011.tar.bz2 › gromacs_4.62_bayesian_saxs_refi/share/html/online/eneconv.html]

eneconv


| eneconv Main Table of Contents |

**VERSION 4.6  
Sat 19 Jan 2013**

---

### Description

With multiple files specified for the -f option:  
Concatenates several energy files in sorted order.
In the case of double time frames, the one
in the later file is used. By specifying -settime you will be
asked for the start time of each file. The input files are taken
from the command line,
such that the command eneconv -f \*.edr -o fixed.edr should do
the trick.

With one file specified for -f:  
Reads one energy file and writes another, applying the -dt,
-offset, -t0 and -settime options and
converting to a different format if necessary (indicated by file
extentions).

-settime is applied first, then -dt/-offset
followed by -b and -e to select which frames to write.

### Files

| option | filename | type | description |
| --- | --- | --- | --- |
| **-f** | ener.edr | Input, Mult. | Energy file |
| **-o** | fixed.edr | Output | Energy file |

### Other options

| option | type | default | description |
| --- | --- | --- | --- |
| **-[no]h** | bool | no | Print help info and quit || **-[no]version** | bool | no | Print version info and quit || **-nice** | int | 19 | Set the nicelevel || **-b** | real | -1 | First time to use || **-e** | real | -1 | Last time to use || **-dt** | real | 0 | Only write out frame when t MOD dt = offset || **-offset** | real | 0 | Time offset for -dt option || **-[no]settime** | bool | no | Change starting time interactively || **-[no]sort** | bool | yes | Sort energy files (not frames) || **-[no]rmdh** | bool | no | Remove free energy block data || **-scalefac** | real | 1 | Multiply energy component by this factor || **-[no]error** | bool | yes | Stop on errors in the file |

### Known problems

- When combining trajectories the sigma and E^2 (necessary for statistics) are not updated correctly. Only the actual energy is correct. One thus has to compute statistics in another way.

---

http://www.gromacs.org  
gromacs@gromacs.org
